# Hippocampal Basal Forebrain Connections Involved in Young Adolescents with Psychotic Experiences

**DOI:** 10.1192/bjo.2025.10215

**Published:** 2025-06-20

**Authors:** Sahar Riaz, Linda Kelly, Michael O’Connor, Mary Cannon, Darren Roddy

**Affiliations:** RCSI, Dublin, Ireland

## Abstract

**Aims:** Changes in the hippocampus and amygdala are associated with psychotic illnesses. However, there is little research examining the output tracts of these regions in psychosis. The fornix connects the hippocampus to the basal forebrain anteriorly and to the hypothalamus posteriorly, while the stria terminalis (ST) connects the amygdala to these same areas. The anterior commissure divides these tracts into anterior (pre-commissural) and posterior (post-commissural) fibres. This study investigates these two tracts and their pre- and post-commissural fibres in young adolescents with psychotic experiences (PEs) as compared with controls across two timepoints (TP), 2 years apart.

**Methods:** 51 young adolescents with PEs (37 female) and 43 healthy controls (25 female) underwent high angular diffusion imaging at TP1, while 39 adolescents with PEs and 29 healthy controls underwent same at TP2. Images were processed using ExploreDTI and, using a bespoke method, the fornix and ST were separated and pre-commissural and post-commissural fibres isolated. Analysis of covariance was performed correcting for age, sex and intracranial volume.

**Results:** Right pre-commissural fornical Mean Diffusivity (MD) (p=0.035) and Radial Diffusivity (RD) (p=0.009) were increased, with decreased Fractional Anisotropy (FA) (p=0.045) at TP1. There was increase across MD (p=0.004), RD (p=0.005) and Axial Diffusivity (AD) (p=0.042) at TP2. Only right pre-commissural fornix MD and RD increases at TP2 survived Bonferroni correction at p=0.0083. No ST differences survived correction for multiple comparisons.

**Conclusion:** This study uses a novel method to separate the stria terminalis and fornix, using an anatomically driven approach. The results show that the hippocampal output fibres are involved in early psychosis, while the amygdala fibres are not affected. Of the hippocampal fibres, it is the fibres going to the basal forebrain, responsible for motivation and behaviour, that are specifically impacted. These changes in adolescents are entirely right sided, reflecting similar right sided hippocampal changes found in adults with psychotic illnesses. The right basal forebrain is known to influence vigilance, attention and emotional processing, which are affected in patients with psychosis. **The findings from this study suggest that the right basal forebrain is affected in children and adolescents with psychotic experiences, which are common in people who go on to develop psychotic illnesses, and thus supports the neurodevelopmental theory of psychosis.**

